# Edible and Medicinal Macrofungi

**DOI:** 10.3390/jof9090908

**Published:** 2023-09-07

**Authors:** Rui-Lin Zhao

**Affiliations:** State Key Laboratory of Mycology, Institute of Microbiology, Chinese Academy of Sciences, Beijing 100101, China; zhaorl@im.ac.cn

**Keywords:** mushroom, phylogeny, biochemistry, cultivation, physiology

## Abstract

Macrofungi are well known as mushrooms, which belong mostly to Basidiomycota with a few from Ascomycota, and up to now, around 40,000 species have been described. In people’s lives, macrofungi are closely related to our economic activities, especially for food and medicine. “One meat, one vegetable and one mushroom” has become a healthy and fashionable dietary structure, and the global edible mushroom production and cultivating area are steadily rising. On the other hand, a large number of mushroom species and new active components have been found, and have become one of the driving forces of innovation of drugs and health products, especially with the development of biochemistry, enzyme engineering, and genetic engineering. Thus, macrofungi in food, medicine, and other aspects have shown a broad prospect. In this Special Issue, research on new species and related molecular phylogenies, mechanisms of hyphae polar growing and basidiocarp formation, biochemistry of edible and medical mushrooms, and some important scientific questions related to the edible mushroom industry are presented, which also reflect the hot areas of common concern on edible and medicinal fungi.

Fungi with large fruiting bodies that can be identified by naked eyes are generally referred to as macrofungi. Macrofungi are distributed almost everywhere, but are especially rich in forests and meadows. Taxonomically, most macrofungi species belong to Basidiomycota with a few from Ascomycota, and up to around 40,000 species have now been described.

In people’s lives, macrofungi are closely related to our economic activities. Humans have a long history of using macrofungi for food and medicine. “One meat, one vegetable and one mushroom” has become a healthy and fashionable dietary structure. From greenhouse cultivation to the construction of a complete edible mushroom industry, the global edible mushroom production and cultivating area are steadily rising. Fungus is a “natural chemical factory”; with the deepening of fungus related research, a large number of species and new active components have been found, and they have become one of the driving forces of innovation of drugs and health products, especially with the development of biochemistry, enzyme engineering, genetic engineering. Thus, macrofungi in food, medicine, and other aspects of life have shown a broad prospect.

In this Special Issue, 16 papers are included, which gather the research achievements on the taxonomy, phylogeny, biochemistry and physiology of edible and medicinal macrofungi (EMM). The main contents can be summarized with the following four topics.

## 1. Discoveries of New Species and Related Molecular Phylogenies

Species of the genus *Russula* (Russulaceae, Russulales) are rich and key components of ectomycorrhizal ecosystems worldwide, some of which are famous edible fungi. In this Special Issue, four new species of *Russula* subsection *Sardoninae* from northern and southwestern China [1] and six new species of *Russula* subgenus *Russula* are described from the Yanshan Mountains in northern Beijing and northern Hebei Province of China [2]. *Hydnobolites* (Pezizaceae, Pezizales) is another ectomycorrhizal fungal genus with hypogeous ascomata. Combining morphological observations with molecular analyses, five new species of *Hydnobolites* were illustrated from Southwest China [3].

## 2. Mechanisms of Hyphae Polar Growing and Basidiocarp Formation 

Although the biochemical and molecular mechanisms of mycelium heterogeneity with polar growing have been studied in many studies, the role of lipids in colony development and zonality is still not understood. In Reference [4], Senik at al. used *Flammulina velutipes* as research material and the heterogeneity in the lipid metabolism and lipid composition of the fungal mycelium was demonstrated [4]. This research on the heterogenicity of the colony will provide the knowledge to explain the processes of hyphal differentiation and mushroom morphogenesis, and also can be used to optimize the yield of the desired lipid metabolite when using fungi as cell factories. 

Basidiocarp formation mechanisms are always interesting. In this Special Issue, the key regulatory pathways of basidiocarp formation of *Pleurotus* spp. were identified through variable methods, such as developing a predictive model, simulation, and system biological analysis methods, in particular using an in silico response to environmental factors and involvement of the major regulatory genes. Overall, cell differentiation and higher expression of respiratory enzymes are the two important steps for basidiocarp formation [5]. *Sanghuangporus baumii* is a traditional medicinal fungus that produces pharmacological terpenoids. The metabolome and transcriptome analysis revealed that four terpenoid hormones dominate the growth and development of this fungi. This study revealed the growth and development mechanisms of *S. baumii* and may promote the breeding and utilization of high-quality varieties [6].

## 3. Biochemistry of Edible and Medical Mushrooms

Edible and medicine mushrooms are highly attractive as they contain abundant bioactive metabolites. *Macrolepiota procera* (MP) is an edible mushroom used in the treatment of diabetes, hypertension, and inflammation. In this Special Issue, the structural features of polysaccharides from *M*. *procera*, in addition to its immunomodulatory activities and effects on probiotic and pathogenic bacteria, are investigated [7]. Another study on polysaccharides from *Volvariella volvacea* (VVP) showed that it could be a good source for a moisturizing, anti-wrinkle, and whitening agent in cosmetic preparations [8].

On one hand, wild edible mushrooms are distributed all over the world and are delicious seasonal foods; on the other hand, they also contain many essential trace elements and are highly enriched in heavy metals, which may be associated with health risks due to exposure to excessive heavy metals in the process of consumption. The contents of four essential trace elements and four harmful heavy metals in nearly 400 species of wild edible mushrooms from 10 countries are reviewed [9]. It was found that the factors affecting the elemental content of edible mushrooms are the difference in species element-enrichment ability, environmental pollution, and geochemical factors. It provides a reference for the risk assessment of edible mushrooms and their elemental distribution characteristics [9]. Another study in this Special Issue aims to determine the mineral content of seven essential metals, Fe, Mg, Mn, P, K, Ca, and Na, in *Lactarius* species collected from southern Spain and northern Morocco. The multivariate study suggested that there were differences between the accumulation of the elements according to the geographic location and species [10].

The role of bioactive metabolites produced by ectomycorrhizal fungi in the establishment of symbiotic relationships is also an interesting scientific question. *Tricholoma vaccinum* is an ectomycorrhizal basidiomycete with high host specificity, and it is able to produce twenty sesquiterpenes. The research showed that three major compounds, Δ^6^-protoilludene, β-barbatene, and an unidentified oxygenated sesquiterpene (*m/z* 218.18), changed production during co-cultivation with the ectomycorrhizal partner tree, *Picea abies*, which could be shown with distinct dynamics. The further research hypothesizes that the sesquiterpene synthase pie1 has an important role during mycorrhization, through Δ^6^-protoilludene and/or its accompanied oxygenated sesquiterpene production [11].

Mushrooms produce a large number of medicinal bioactive metabolites with antioxidant, anticancer, antiaging, and other biological activities. However, whether they produce flavonoids and, if so, how they synthesize them remains a matter of some debate. The authors combined flavonoid-targeted metabolomics and transcriptome analysis to reveal that *Sanghuangporus baumii* synthesized 81 flavonoids on a chemically defined medium. Further analysis suggests that the flavonoid synthesis pathway in *S. baumii* is different from that in known plants, and the missing genes may be replaced by genes from the same superfamilies but that are only distantly related. This study provides a novel method to produce flavonoids via metabolic engineering using mushrooms [12].

## 4. Some Important Scientific Questions Related to the Edible Mushroom Industry

The enoki mushroom (*Flammulina filiformis*) is one of the most important and popular edible mushrooms commercially. However, traditional mushroom cultivar identification is challenging due to poor accuracy, heavy workloads, and low reproducibility. To overcome this challenge, the authors developed a method for identifying *F. filiformis* strains using multiple nucleotide polymorphism sequencing (MNP-seq) [13]. 

True morels (*Morchella*, Pezizales) are world-renowned edible mushrooms that are widely demanded in international markets. However, nearly 25% of the total cultivation area has annually suffered from fungal diseases. In this paper, it is shown that *D. longispora* is a major culprit of morel fungal diseases through wide investigation and critical identification [14].

*Agaricus subrufescens* is well known worldwide due to its highly medicinal and nutritional properties. A work from this Special Issue evaluated the bacterial community present in mushroom-colonized compost extract (MCCE) prepared from cultivation of *A. subrufescens*. This work showed the dynamics with two different soaking times and the influence on the biological efficiency, precociousness, and mushroom weight, which could provide new strategies to enhance the yield and quality of *A. subrufescens* cultivation [15]. 

Soil origin, mycorrhizal plant partners, and environmental factors affect the growth and development of SongRong (*Tricholoma matsutake*). In order to clarify the relationships of fungi–bacteria networks and various influence factors in the habitat of SongRong, high-throughput sequencing and analysis revealed fungal–bacterial networks in the habitat of SongRong and driving factors of their distribution rules [16].

In general, the papers presented in this Special Issue also reflect the hot areas of common concern in edible and medicinal fungi, representing the development trends of the area. We hope that more studies will emerge in the second edition of the Special Issue Edible and Medicinal Macrofungi in the close future.
